# It’s not just droplets: a systematic review and meta-analysis of the modes of transmission of Group A *Streptococcus*

**DOI:** 10.3389/fpubh.2025.1630054

**Published:** 2025-09-04

**Authors:** Dylan D. Barth, Jessica Daw, Stephanie L. Enkel, Tracy McRae, Jonathan R. Carapetis, Rosemary Wyber, Asha C. Bowen, Mark E. Engel

**Affiliations:** ^1^Wesfarmers Centre of Vaccines and Infectious Diseases, Telethon Kids Institute, Perth, WA, Australia; ^2^Centre for Child Health Research, The University of Western Australia, Perth, WA, Australia; ^3^Department of Infectious Diseases, Perth Children’s Hospital, Perth, WA, Australia; ^4^The George Institute for Global Health, Sydney, NSW, Australia; ^5^AFROStrep Registry, Department of Medicine, The University of Cape Town, Cape Town, South Africa; ^6^SA Cochrane Centre, South African Medical Research Council, Cape Town, South Africa

**Keywords:** Group A *Streptococcus*, systematic review, environmental health, infectious diseases, meta-analysis, transmission, primordial prevention, epidemiology

## Abstract

**Background:**

The transmission of Group A *Streptococcus* (Strep A) through respiratory droplets has been considered the dominant mode of transmission to date; however, little is known about the relative contribution of other modes of transmission. This review systematically summarises the contemporary evidence regarding the transmission of Strep A.

**Methods:**

A comprehensive search strategy was implemented to identify studies on Strep A transmission published in English between 1980 and 2019. Full-text articles were screened and included based on the predefined criteria. Studies were included if molecular techniques were used to identify the same Strep A strain in both clinical and environmental swabs. A random-effects meta-analysis model was used to aggregate attack rate estimates with 95% confidence intervals (CI), incorporating the Freeman–Tukey transformation to account for variability between studies.

**Results:**

A total of 34 transmission cohorts were included in this study. The overall attack rate of Strep A was 18.4% (95% CI, 13.1–24.2%, I^2^ = 95.9%), for direct contact, it was 20.5% (95% CI, 8.3–35.4%), and for indirect contact, it was 19.1% (95% CI, 13.2–25.7%). When pooled by geographical location, the attack rate was 30.38% (95% CI, 20.89–40.75%) in non-urban settings and 7.36% (95% CI, 2.60–14.21%) in urban settings.

**Conclusion:**

Direct contact is no longer the dominant form of Strep A transmission. Our contemporary findings have implications for the development of evidence-based environmental health strategies aimed at reducing Strep A transmission.

**Systematic review registration:**

https://www.crd.york.ac.uk/PROSPERO/view/CRD42019138472, CRD42019138472.

## Introduction

Group A *Streptococcus* (Strep A) is responsible for a wide range of diseases and is ranked globally among the top 10 pathogens causing morbidity and mortality, particularly in low-income settings (1). Strep A causes superficial infections (pharyngitis, impetigo, and scarlet fever), invasive infections (cellulitis, skeletal infections, sepsis, necrotising fasciitis, and toxic shock syndrome), and post-infectious immune-mediated sequelae (acute rheumatic fever (ARF)/rheumatic heart disease (RHD) and post-streptococcal glomerulonephritis). The global prevalence of RHD is estimated to be 33 million cases, leading to approximately 319,000 deaths each year (2). To date, a vaccine to prevent Strep A infection remain elusive (3).

The transmission of Strep A has historically been attributed to large respiratory droplets (4). This conclusion is based on studies that employed one of two methods: (a) analysing the saliva of patients with Strep A sore throat and scarlet fever and (b) conducting environmental sampling to measure the amount of Strep A released into the air in a controlled room through actions such as coughing, sneezing, and talking. These studies have predominantly focused on gathering knowledge to inform infection control activities within healthcare or communal settings, such as military barracks, rather than adopting a broader community-wide perspective to reduce the global burden of Strep A infections and their sequelae. More recently, a combination of methodological approaches has been used to explore the possibility of transmission via direct contact. These methods include culturing samples from biological swabs, conducting environmental surface swabs, and utilising environmental settle plates. These studies have elucidated additional modes of Strep A transmission, including airborne transmission (5).

Transmission-based precautions are a crucial component of infection control (6), particularly in healthcare settings. However, it is equally important to understand the modes of transmission of Strep A infection within households and communities for developing strategies that focus on interrupting these modes of exposure to reduce the risk of infection and related diseases. Thus, we aimed to (1) synthesise evidence on modes of transmission for Strep A, (2) calculate and compare attack rates by mode of transmission, and (3) correlate, when possible, the *emm* types of Strep A isolated from clinical and environmental swabs with the respective mode of transmission. We also explored the impact of contextual and environmental settings on Strep A transmission.

## Methods

### Search strategy and selection criteria

This systematic review followed the Preferred Reporting Items for Systematic Reviews and Meta-Analyses (PRISMA) guidelines, and the protocol was published and registered with PROSPERO (CRD42019138472), which details the inclusion and exclusion criteria, search strategy, and screening and selection processes (7). Articles that were not suitable for inclusion in the meta-analysis were initially intended for narrative discussion. However, due to the substantial number of articles identified, a separate literature review can be compiled for subsequent publications.

The search strategy involved using terms such as “*Streptococcus*,” “transmission,” “outbreak,” and “infection” in PubMed, Scopus, EMBASE, Web of Science, and Cumulative Index to Nursing and Allied Health Literature (CINAHL) databases. The search focused on English-language studies published between 1980 and 31 December 2019. Unpublished studies, grey literature, and preprints were also included in the review. We excluded studies published after 2019 due to the confounding influence of the pandemic, which may not have provided sufficient detail regarding non-pharmaceutical interventions such as masking, lockdowns, and handwashing.

Outcome measures included documented evidence of Strep A transmission through different modes and attack rates of individuals with Strep A infection (symptomatic) and detection (asymptomatic carriage) in an exposed population. Two attack rates were calculated: (a) the ‘probable attack rate’ based on clinical symptoms and (b) the ‘confirmed attack rate’ based on DNA analysis via molecular typing (e.g., M and T proteins) or visual identification methods (e.g., pulsed-field gel electrophoresis (PFGE), randomly amplified polymorphic DNA (RAPD), and multilocus sequence typing (MLST)). The attack rates for each transmission mode were calculated and compared with one another.

We included studies examining routes of transmission, categorising them according to direct or indirect transmission pathways, as described in the original publications, following the criteria defined by Bonita et al. (8). The studies included in this review were categorised based on the described mode of Strep A transmission, as summarised in Table 1.

**Table 1:** Classification of transmission mechanisms for Strep A.

Transmission type	Mode	Description	Examples (for Strep A)
Indirect	Airborne	Infectious agents carried by fine expectorated sputum or dust suspended in the air.	Fine droplets/aerosols in air containing Strep A.
	Vehicles	Inanimate objects that can transmit an infectious agent (fomites).	Contaminated surfaces, bedding, fabrics, food, or medical equipment.
	Vectors	Living organisms that carry infectious agents through mechanical or biological means.	Flies, mosquitoes, fleas, or animals carrying Strep A.
Direct	Droplets	Transmission through droplets produced by sneezing, coughing, or talking.	Nasal secretions, sputum, or saliva containing Strep A.
	Contact	Physical contact where the pathogen is in secretions such as pus or serous fluid.	Skin-to-skin contact transferring Strep A through infected pus or fluid.

### Data synthesis

We documented the molecular techniques used to confirm the mode of Strep A transmission. The risk of bias was assessed independently using the Critical Appraisal tool from the Joanna Briggs Institute (9). To address the appropriateness of the statistical analysis used in each study, we assessed whether the numerator and denominator were adequately reported to calculate the attack rate. The data were reported according to the modes of transmission.

To accurately measure transmission and eliminate chance findings, studies were only included if molecular techniques were applied and if the same Strep A strain was identified in both clinical and environmental swabs. Data were analysed using STATA version 16 (StataCorp, College Station, TX, USA). We calculated attack rates and standard errors (SE) and subjected the data to meta-analysis (random-effects model, due to the expected variability across the studies), using the Metaprop_one package (overall estimate with 95% confidence interval (CI)). The pooled rates were estimated using the Freeman–Tukey double arcsine transformation method to stabilise the variance of attack rates within each study (10).

Heterogeneity was assessed using the *I*^2^ heterogeneity statistic and reported as a percentage, as defined by Deeks: ≤25% low, 26–50% moderate, 51–75% substantial, and 76–100% considerable heterogeneity (11). Where heterogeneity was statistically significant, a sensitivity analysis was conducted to explore the potential source, e.g., the quality of the studies (risk of bias) or sample size. Where available, a subgroup analysis was conducted according to study setting (nosocomial, domestic, and public settings) and geographical regions.

## Results

**Figure 1:**
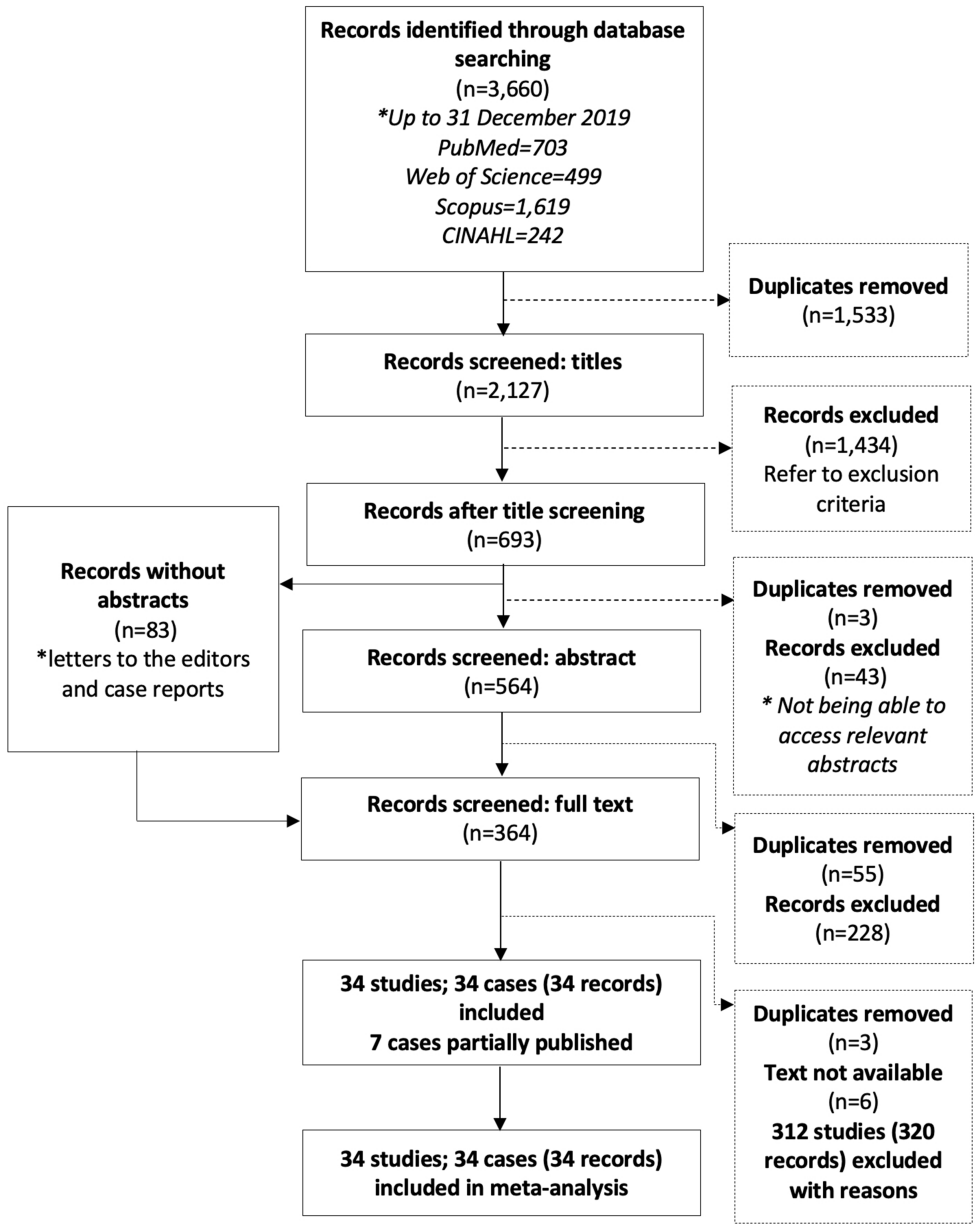
Flow diagram of search results and screening process.

From the searched databases, 3,660 records were retrieved, with 2,127 remaining after duplicates were removed (Figure 1). After title and abstract screening, 364 full-text records were assessed for inclusion.

A total of 34 studies comprising 34 different transmission cohorts met the inclusion criteria for this review. Reasons for exclusion included lack of data suitable for analysis (123 records; 120 studies), outbreak summary without transmission routes mentioned (69 records; 64 studies), narrative reviews and articles lacking primary outbreak data (48 records; 48 studies), summaries of infection trends (44 records; 44 studies), lab-based research (23 records; 23 studies), symptom/diagnostic method/treatment (9 records; 9 studies), and microorganisms other than Strep A (3 records; 3 studies).

### Studies included in the meta-analysis

**Figure 2:**
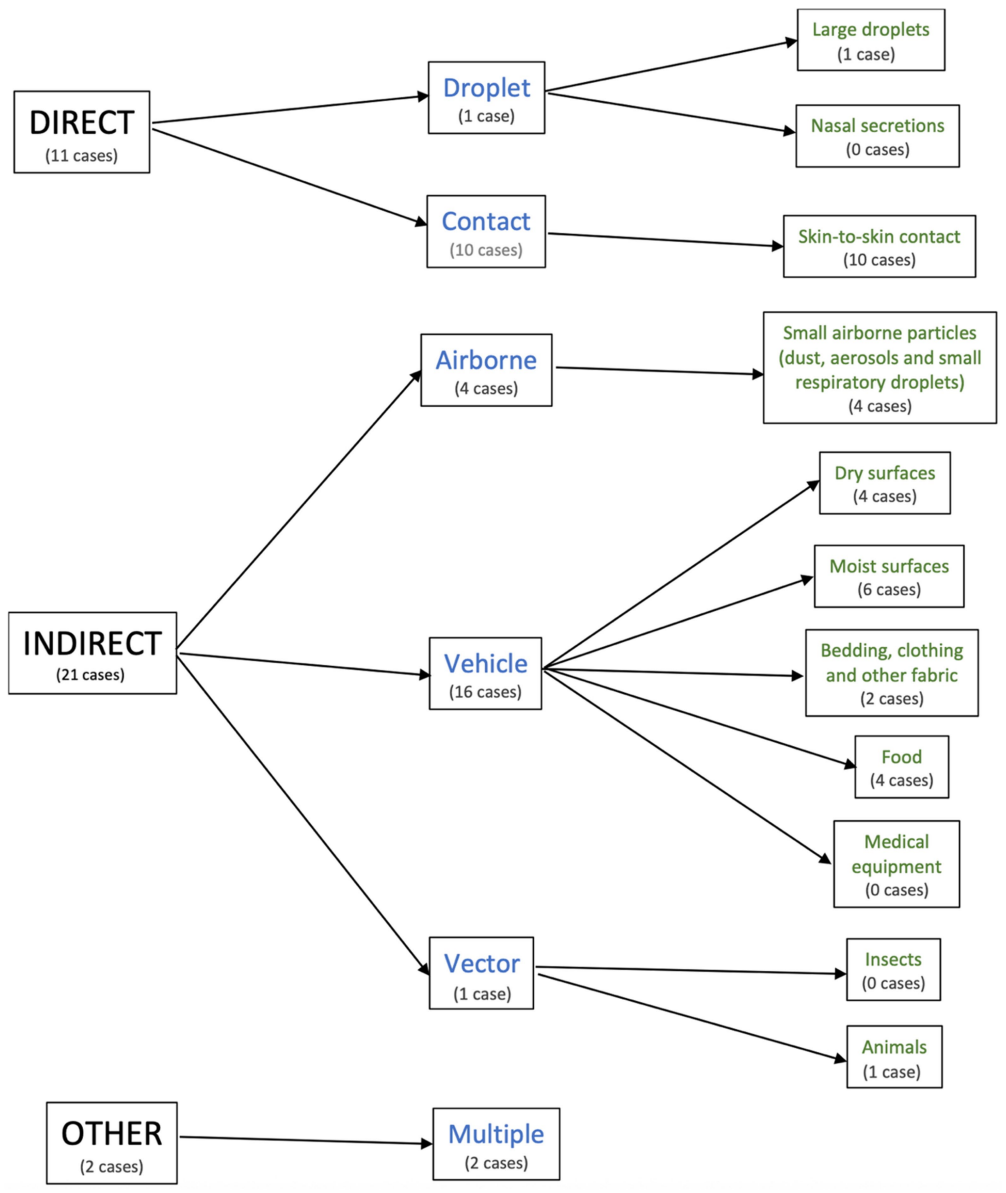
Summary of the modes of Strep A transmission in the included cohorts.

The characteristics of the included studies are summarised in Supplementary Table 1. The transmission cohorts were categorised based on their mode of transmission (Figure 2). Of these, 11 were direct (droplet, *n* = 1; contact, *n* = 10), 21 were indirect (airborne, *n* = 4; vehicle, *n* = 16; vector, *n* = 1), and 2 were multiple (airborne and vehicle; *n* = 2).

All included studies provided data suitable for meta-analysis, as they incorporated DNA methods for the confirmation of transmission. The study designs included cohort (*n* = 15), case–control (*n* = 6), cross-sectional (*n* = 6), and case report studies (*n* = 7). These studies were conducted in Europe (*n* = 18), North America (*n* = 12), Asia (*n* = 2), Oceania (*n* = 1), and the Middle East (*n* = 1). The median number of people exposed in each transmission cohort for whom data were available (*n* = 34) was 73 (interquartile range [IQR], 21–225). Of these, 55.9% reported <100 exposed cases, 29.4% reported 100–500 cases, and 14.7% reported >500 cases. Urban (*n* = 21) and non-urban (*n* = 6) environments were identified, seven of which were not stated. The most common settings were nosocomial (*n* = 14) and public (*n* = 16), with four occurring within the household.

The majority of transmission cohorts involved superficial infections, 24 out of 34 cohorts (70.5%), with animals exclusively associated with impetigo and pharyngitis. There were 4 out of 34 (11.7%) transmission cohorts that also reported invasive Strep A infections. Contaminated medical equipment was exclusively associated with invasive infections.

All 34 transmission cohorts were pooled to determine the overall Strep A attack rate. There were 11 cohorts with direct transmission (droplet, *n* = 1; contact, *n* = 10) and 21 cohorts with indirect transmission (airborne, *n* = 4; vehicle, *n* = 16; and vector, *n* = 1). The remaining two cohorts were assigned to the category “other” (multiple, *n* = 2). All transmission cohorts belonged to the following modes of transmission: large droplets (*n* = 1); skin-to-skin contact (*n* = 10); small airborne particles (dust, aerosols, and small respiratory droplets, *n* = 4); dry surfaces (*n* = 4); moist surfaces (*n* = 6); bedding, clothing, and other fabric (*n* = 2); food (*n* = 4); animals (*n* = 1); and multiple modes (*n* = 2). None of the cohorts was attributed to nasal secretions, medical equipment, or insects.

**Figure 3:**
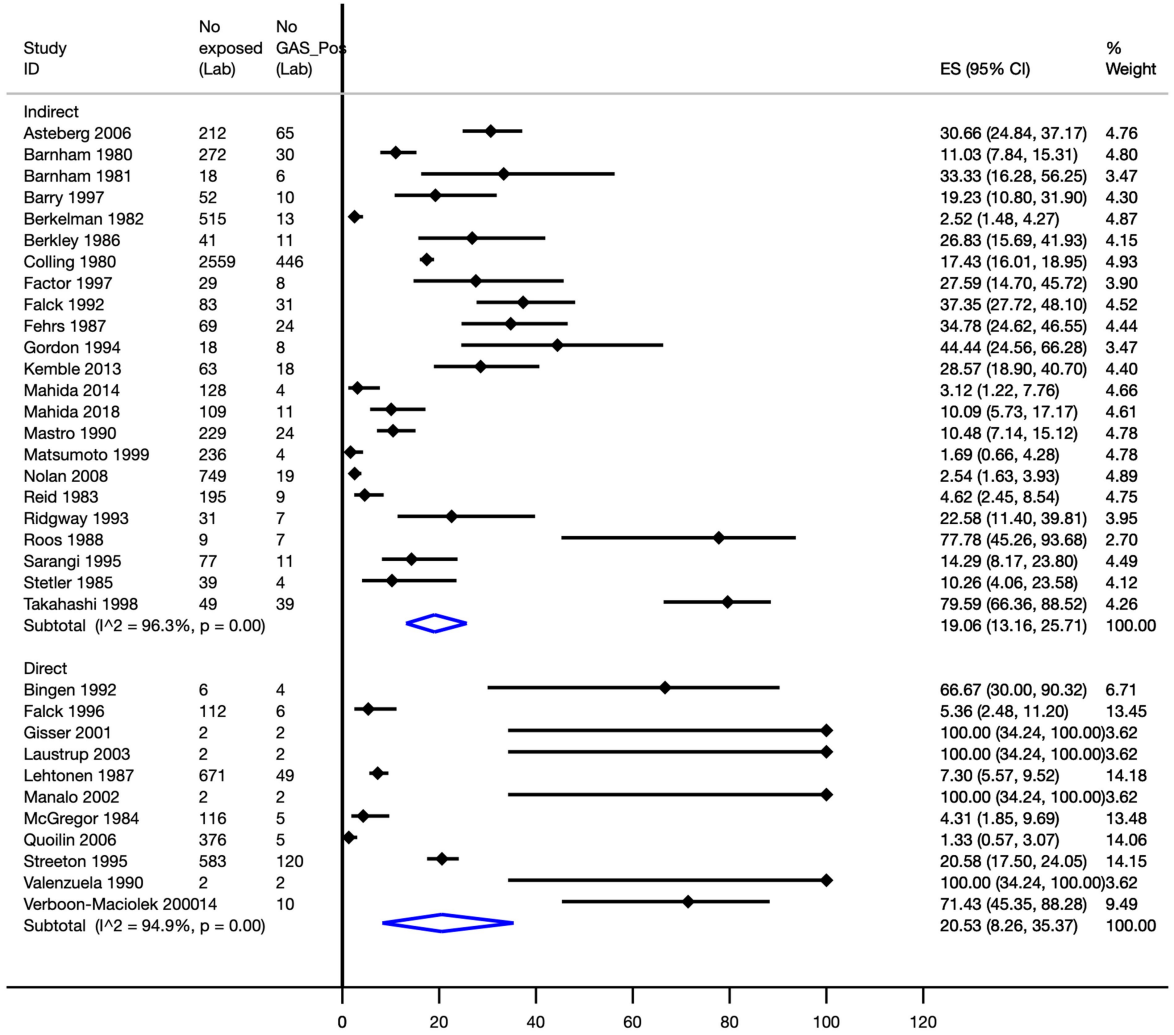
Strep A’s attack rate by type of contact. ES, effect size; CI, confidence interval.

The overall attack rate was 18.4% (95% CI, 13.1–24.2%; 34 transmission cohorts, *n* = 7,668; I^2^ = 95.7%). A subgroup analysis to evaluate the attack rate by direct and indirect contact determined, for indirect transmission, a pooled attack rate of 19.1% (95% CI, 13.2–25.7%; I^2^ = 96.3%), whilst from studies of direct transmission, a pooled attack rate of 20.5% (95% CI, 8.3–35.4%; I^2^ = 94.9%) was observed (Figure 3).

**Figure 4:**
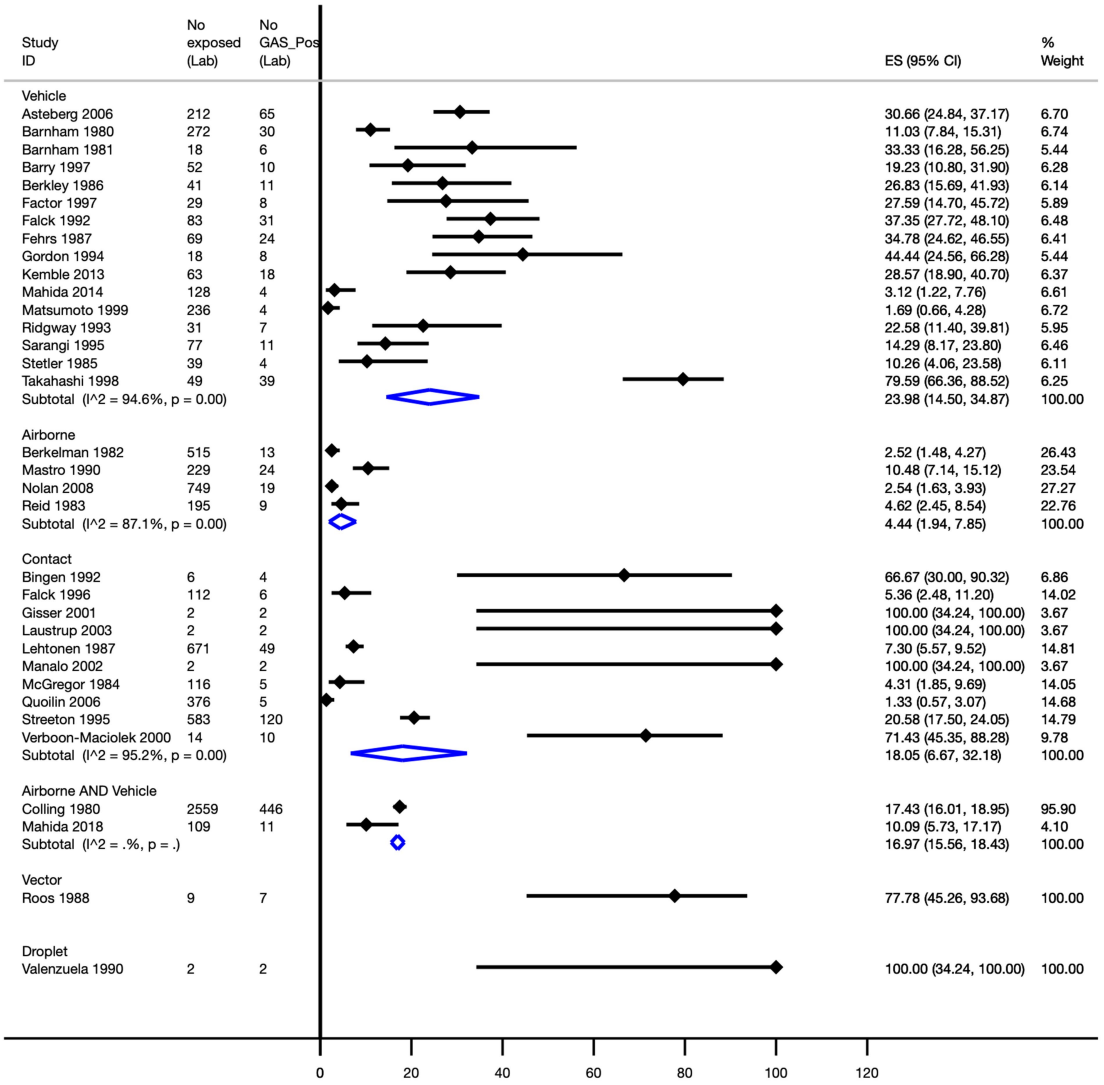
Pooled Strep A attack rate by mode of transmission. ES, effect size; CI, confidence interval.

Pooled attack rates were calculated as follows: vehicle, 24.0% (95% CI, 14.5–34.9%); direct contact, 18.1% (95% CI, 6.7–32.2%); airborne, 4.4% (95% CI, 1.9–7.9%); and a combination of airborne and vehicle-mediated modes of transmission, 17.0% (95% CI, 15.6–18.4%). A single study reported attack rates of 77.8% (95% CI, 45.3–93.7%) for vector-mediated transmission and 100% (95% CI, 34.2–100.0%) for droplet-mediated transmission (Figure 4).

**Figure 5:**
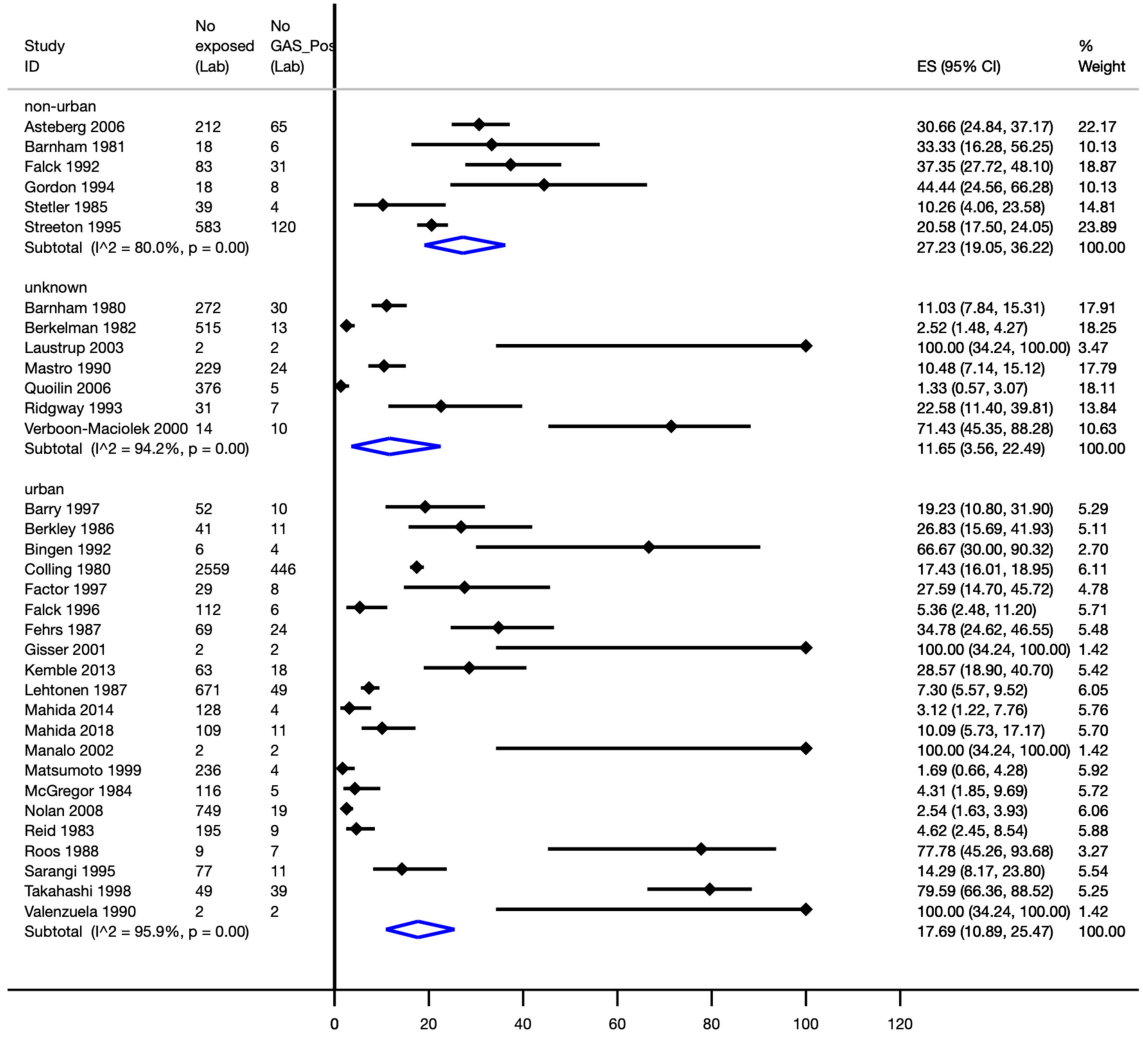
Pooled Strep A attack rate according to the geographical setting. ES, effect size; CI, confidence interval.

The pooled Strep A attack rates in non-urban and urban geographical settings were 27.2% (95% CI, 19.1–36.2%) and 17.7% (95% CI, 10.9–25.5%), respectively (Figure 5).

No discernible pattern of transmission was observed, with combined rates ranging from 11.1% (95% CI, 1.1–27.8%) in autumn (*n* = 4) to 23.1% (95% CI, 10.4–38.8%) in studies that did not delineate seasons of transmission (*n* = 6). Winter and spring had pooled rates of 12.5% (95% CI, 3.7–24.9%; I^2^ = 91.3%) (*n* = 5) and 15.9% (95% CI, 2.7–34.8%; I^2^ = 96.7%) (*n* = 5).

The pooled Strep A attack rates by non-urban and urban geographical settings among high-quality studies were 30.4% (95% CI, 20.9–40.8%) and 7.6% (95% CI, 2.6–14.2%), respectively; this difference was statistically significant (*p* < 0.0001).

The risk of bias for the 34 cohorts was assessed for each category to give a total score of 10, with 10 indicating a low risk of bias. The risk of bias for each category of the meta-analysis cohort is shown in Supplementary Table 2. The evaluation covered key domains relevant to the robustness of reporting and internal validity, including whether studies had clearly defined inclusion criteria, whether the condition (Strep A infection) was measured in a standard and reliable manner for all participants, and whether valid methods were used to identify the condition. Additional criteria assessed whether participant inclusion was consecutive and complete, whether demographic and clinical information was clearly reported, whether outcomes and follow-up results were transparently presented, and whether the study site was adequately described. The appropriateness of statistical analyses was also considered. The results of this quality appraisal informed the overall synthesis by allowing greater interpretive weight to be assigned to studies with a lower risk of bias. Variations in study quality were accounted for when interpreting the findings on the transmission mechanisms and attack rates. Two cohorts (5.9%) had scores between 0 and 4, 19 cohorts (55.9%) had scores between 5 and 7, and 12 cohorts (35.3%) had a low risk of bias with a score between 8 and 10. One cohort was not applicable to the risk of bias scores, as it was partially published with limited information.

## Discussion

This study presents a comprehensive review of Strep A transmission and provides the first quantification of attack rates based on transmission modes from laboratory-confirmed data. There are three important findings.

1. Strep A attack rates were high for both direct and indirect modes of transmission. Whilst this was expected for direct transmission based on conventional wisdom, the finding that indirect transmission was equally high was a novel finding,
2. Vector-mediated modes of transmission were a surprising finding, highlighting the possible role of animals in Strep A transmission,
3. Strep A attack rates in non-urban settings were higher than those in urban settings.

Our systematic review confirmed that droplet transmission is an important modality for Strep A. In contrast to conventional wisdom, there is a range of other transmission modes that are important for controlling Strep A disease. Although droplet transmission is only one of the many transmission modalities, it has the highest reported attack rate of 100%, albeit from only one study. Non-pharmaceutical interventions, including social distancing, staying home when unwell, wearing masks, catching coughs and sneezes in the elbow, and hand hygiene, remain important strategies to reduce the transmission of Strep A. This may explain why Strep A-related diseases were far lower during the COVID-19 pandemic years, with a surge commencing worldwide in late 2022 (12). In addition, other modes of transmission were even more common but with lower attack rates, e.g., vector-mediated, contact, and airborne. Strategies for Strep A control have become more complex, with these nuanced mechanisms requiring attention to ventilation, vermin control, and surface cleaning. Airborne-mediated transmission of Strep A had the lowest attack rate at 4.4% (*n* = 4 cohorts). However, Strep A was not previously thought to have an airborne transmission route. From these studies, it is clear that the transmission of Strep A is multimodal and that this pathogen is highly infectious to close contacts in an index case. This contextual information is important to inform the development of contemporary control strategies for Strep A in the hospital, community, and household setting, whilst the development of a vaccine progresses (SAVAC).^1^

The COVID-19 pandemic has prompted a fundamental re-evaluation of respiratory pathogen transmission models. Early in the pandemic, public health strategies were heavily focused on surface cleaning and physical distancing based on the assumption that SARS-CoV-2 spread primarily through large respiratory droplets. However, as evidence emerged supporting aerosol transmission, attention shifted towards airborne spread, prompting the widespread adoption of masks as well as increased emphasis on indoor air quality, ventilation, and crowding reduction (13, 14). This evolving understanding highlights the limitations of the traditional droplet-airborne dichotomy and underscores the importance of environmental and situational factors. Our review of Strep A transmissions suggests a similar need for further conceptual broadening. Whilst Strep A has long been considered primarily for droplet spread, emerging evidence points to the role of indirect contact, fomite transmission, and possibly vector spread under specific conditions. These findings align with the broader recognition that transmission should be viewed as a continuum rather than a binary model, an approach that could strengthen infection prevention strategies for Strep A and other respiratory pathogens.

Before the mid-twentieth century, severe and post-infectious Strep A events were common to all populations. With improvements in living conditions, the burden of these diseases has declined in most high-income countries, but the same gains have not been shared equally. There is an urgent need for contemporary environmental health initiatives to reduce Strep A transmission and its downstream complications, especially in close living environments and overcrowded households at a high risk of Strep A-related sequelae. This review provides novel insights into the design of such initiatives.

Strep A has been cultured from a range of environmental surfaces, including dry surfaces such as door handles, bench surfaces, and plastic toys (15–19); fabrics such as carpets, curtains, and soft furnishings (17, 20, 21); and moist surfaces such as bidets, tap handles, toothbrushes, and chewed pencils (16, 17, 22–24). Strep A can persist on dry inanimate surfaces for up to six months and in liquid culture conditions for up to a year (25). A study examining the long-term survival of Strep A observed enhanced tolerance of Strep A in desiccated conditions, suggesting that environmental surfaces may be an important source of transmission and reinfection (26). The compilation of these results is powerful for identifying potential environmental health avenues that are now shown to be relevant to Strep A transmission. Recognition of the risk of cross-infection with shared toothbrushes is also important.

In addition to environmental surfaces, foodborne disease transmission has also been implicated. Strep A is cultured from food items such as egg-based products and other leftover foods (27–30). Efforts to prevent foodborne transmission of Strep A should consider the context in which there are challenges in maintaining the cold chain and where food may be stored for prolonged periods before consumption.

Strep A has also been isolated from small airborne particles in a variety of hospital areas and communal living spaces, such as dormitories (21), as well as from medical equipment, including intrauterine contraceptive devices (31).

Our sensitivity analysis of a subset of studies deemed high quality showed a significant difference with respect to attack rates between studies conducted in non-urban settings (30.0%) and studies in urban settings (7.0%). Whilst this difference may be limited by publication bias, a likely explanation may be that the social determinants of health are exacerbated in non-urban settings.

Whilst a number of historic studies investigated the possibility of Strep A contamination spread through dust in the household or hospital environment (4), we only found one contemporary piece of evidence of a Strep A positive dust sample, which came from underneath beds (17). However, the authors of this study proposed that transmission in this cohort occurred via moist surfaces.

Strep A has been widely considered a human-only pathogen; however, this systematic review found Strep A isolated in three transmission cohorts involving three animals: two in cats (throat and eye) and one in a dog’s eye (16, 32). In all three transmission cohorts, molecular methods were used to identify the same Strep A strains in both human and animal swabs. One of the three studies clearly established the direction of transmission and thus was included in the meta-analysis (16, 32). The study described recurrent tonsillitis among family members, which resolved only following treatment of their Strep A-positive cat, thereby identifying the cat as the source of infection, with a reported human attack rate of 77.8% (16, 32). In contrast, the remaining two studies involving two separate families detected Strep A in the conjunctiva of household pets: a cat in one case and a dog in the other, but were unable to establish evidence of animal-to-human transmission. In one household, the dog was reported to sleep in the beds of family members, whereas in the other, the cat was frequently handled by children in the family nursery. Despite the lack of confirmed directionality, the authors highlighted the potential for animal-to-human transmission given the close nature of interactions with humans and their domestic animals (16, 32). Although the total number of people exposed to animals as a vector was low (*n* = 17), these studies utilised molecular techniques to confirm that the same strain was isolated from both animal and human samples. This is also supported by an earlier study that implicated household pets as the source of recurrent pharyngitis in pet owners’ children after isolation (33). These animal findings were surprising, in contrast to screening studies for Strep A in animals. In the Queensland Aboriginal community, dogs were tested for the presence of Strep A, where throat swabs of the community (*n* = 57) and wild dogs (*n* = 4) were collected, including dogs from households with Strep A skin infections. No Strep A was detected in dogs (34). In the United States, household pets of children with and without confirmed Strep A throat infections were tested for Strep A within 72 h of infection. Cultures were collected from the throats of all cats and dogs and from other sites in a subset of animals. No Strep A was identified on any of the 452 samples from 230 animals (35). In contrast, in a recent study that involved domestic dogs and cats being admitted to a veterinary hospital with symptoms of respiratory illness, nasal and oral swabs were obtained and molecular methods were used to confirm the presence of Strep A (36). This study reported Strep A prevalence in symptomatic dogs and cats to be 15.0 and 7.0%, respectively, with a small proportion of macrolide-resistant strains in both. This study highlights a possible role of animals in the epidemiological infection cycle. In another study, Strep A was isolated from the skin and genital tract specimens of animals (37). Whilst these studies indicate the ability of human-derived Strep A to colonise and infect animals, the extent to which animals should be considered a *new* reservoir of Strep A requires contemporary research to confirm animal-associated transmission to humans, challenging the notion that Strep A is a human-only pathogen.

We found no studies providing evidence of insect-associated transmission that met the inclusion criteria of our review. Some laboratory studies suggest that house flies may be able to digest and excrete live Strep A bacteria (38). Additionally, there is no evidence that bedbugs are vectors for transmitting Strep A infections (39). However, it is likely that biting insects are a risk factor for minor skin damage, which may lead to opportunistic bacterial infections.

We did not identify any cohorts that suggested nasal transmission of Strep A infection, despite the high prevalence of respiratory Strep A infections. In one study of children across 12 remote Aboriginal communities, 7.0% of children with skin infections also had Strep A in the anterior nares (40). However, given that nasal discharge is common among children, this small proportion could have a significant impact on transmission.

One of the strengths of our systematic review is that it distinguished between probable and confirmed Strep A infections, which provided robust and reliable data to quantify attack rates. To confirm the transmission mechanism, Strep A must be confirmed by molecular typing or visual identification to determine the similarity between the Strep A strains that cause an outbreak. This allows for a high degree of certainty in our data, providing the first attempt at quantifying attack rates associated with transmission mechanisms and summarising Strep A. Furthermore, our large sample size (>7,500 people exposed) included in the meta-analysis covered a variety of countries, time periods, seasons, and types of Strep A infections.

There were a number of limitations in our systematic review of the studies included in the meta-analysis. Given the requirement for studies using molecular typing or visual identification methods for Strep A, the attack rates we calculated may underrepresent the true rates of infection, because some cases do not yield culturable or typable samples (especially cellulitis), or mild cases do not come to light. The authors may also potentially not have a budget for typing all isolates or any at all, and techniques for strain sequencing may have evolved over the study period. This creates a possible temporal bias, with greater confidence in more recent studies. Furthermore, the reporting of outbreaks in general was poor despite the heavy burden of Strep A globally. It is often difficult to ascertain the number of people exposed to and with Strep A infections. This is demonstrated in the risk of bias, with only 12/34 cohorts (35.3%) having a good score (8–10). Publication bias may also contribute to this, as outbreak investigations are not always published. Similarly, there may be difficulties in identifying outbreaks because exposed individuals may present to different health professionals, where swabbing is not a standard practise (41). Furthermore, unlike within the hospital environment, familial or household cases or public places such as day care centres are not usually swabbed. Finally, the high heterogeneity observed (and somewhat expected) could not be explained by sensitivity analyses.

The findings of this review have implications for public health responses aimed at reducing Strep A transmission. Specific clinical and public health recommendations are beyond the scope of this review and should be situated in a future contextual analysis of the current guidance and suggested revisions. This should include the consideration of equity and implementation outcomes.

## Conclusion

To our knowledge, this review is the first to systematically synthesise the transmission mechanisms and attack rates of Strep A. Our evidence indicates that the traditional attribution of large respiratory droplets as the primary mode of spread may be imprecise, and consideration must be given to additional modalities, including environmental reservoirs, vectors, and airborne routes. Furthermore, this review highlighted that animal transmission warrants further investigation and that contacts in household and classroom settings may be at the highest risk of human-to-human transmission. This study provides novel insights and evidence for environmental health and prevention strategies to disrupt transmission mechanisms.

## Data Availability

The original contributions presented in the study are included in the article/Supplementary material, further inquiries can be directed to the corresponding author.

## Footnotes

^1^ https://savac.ivi.int/
