# Supplementary material for: It’s not just droplets: a systematic review and meta-analysis of the modes of transmission of Group A Streptococcus
Source: Front Public Health. 2025 Sep 4;13:1630054. doi: 10.3389/fpubh.2025.1630054 (PMC12443793; doi:10.3389/fpubh.2025.1630054)
Supplement: Supplementary file 1 [file Supplementary_file_1.docx]

**Supplementary Table 1: Characteristics of included studies**

| **Transmission cohort** | **Study reference** | **Study design** | **Transmission category** | **Transmission mode** | **Study setting** | **Country** | **Geographical setting** | **Strep A attack rate (%)** |
| --- | --- | --- | --- | --- | --- | --- | --- | --- |
| 1 | Asteberg et al., 2006 [1] | Cohort study | Vehicle | Food | Public | Europe | Non-urban | 30.66% |
| 2 | Barnham et al., 1980 [2] | Cross sectional study | Vehicle | Moist surfaces | Public | Europe | Unknown | 11.03% |
| 3 | Barnham et al., 1981 [3] | Cross sectional study | Vehicle | Moist surfaces | Public | Europe | Non-urban | 33.33% |
| 4 | Barry et al., 1997 [4] | Cohort study | Vehicle | Dry surfaces | Public | North America | Urban | 19.23% |
| 5 | Berkelman., 1982 [5] | Case control study | Airborne | Small airborne particles (dust, aerosols, small respiratory droplets) | Nosocomial | North America | Unknown | 2.52% |
| 6 | Berkley et al., 1986 [6] | Case control study | Vehicle | Food | Public | North America | Urban | 26.83% |
| 7 | Bingen et al., 1992 [7] | Case report | Contact | Skin-to-skin contact | Nosocomial | Europe | Urban | 66.67% |
| 8 | Colling et al., 1980 [8] | Cohort study | Multiple | Multiple: Small airborne & bedding, fabrics | Public | Europe | Urban | 17.43% |
| 9 | Factor et al., 1997 [9] | Cohort study | Vehicle | Dry surfaces | Public | North America | Urban | 27.59% |
| 10 | Falck et al., 1992 [10] | Case control study | Vehicle | Moist surfaces | Public | Europe | Non-urban | 37.35% |
| 11 | Falck et al., 1996 [11] | Cross sectional study | Direct | Skin-to-skin contact | Public | Europe | Urban | 5.36% |
| 12 | Fehrs et al., 1987 [12] | Cohort study | Vehicle | Moist surfaces | Public | North America | Urban | 34.78% |
| 13 | Gisser et al., 2001 [13] | Case report | Contact | Skin-to-skin contact | Domestic | Asia | Urban | 100.00% |
| 14 | Gordon et al., 1994 [14] | Cross sectional study | Vehicle | Moist surfaces | Nosocomial | Europe | Non-urban | 44.44% |
| 15 | Kemble et al., 2013 [15] | Cohort study | Vehicle | Food | Public | North America | Urban | 28.57% |
| 16 | Laustrup et al., 2003 [16] | Case report | Contact | Skin-to-skin contact | Domestic | Europe | Unknown | 100.00% |
| 17 | Lehtonen et al., 1987 [17] | Cohort study | Direct | Skin-to-skin contact | Nosocomial | Europe | Urban | 7.30% |
| 18 | Mahida et al., 2014 [18] | Cohort study | Vehicle | Bedding, clothing and other fabric | Nosocomial | Europe | Urban | 3.13% |
| 19 | Mahida et al., 2018 [19] | Cohort study | Multiple | Multiple: Small airborne & bedding, fabrics | Nosocomial | Europe | Urban | 10.09% |
| 20 | Manalo et al., 2002 [20] | Case report | Contact | Skin-to-skin contact | Domestic | North America | Urban | 100.00% |
| 21 | Mastro et al., 1990 [21] | Case control study | Airborne | Small airborne particles (dust, aerosols, small respiratory droplets) | Nosocomial | North America | Unknown | 10.48% |
| 22 | Matsumoto et al., 1999 [22] | Cross sectional study | Vehicle | Food | Public | Asia | Urban | 1.69% |
| 23 | McGregor et al., 1984 [23] | Case control study | Contact | Skin-to-skin contact | Nosocomial | North America | Urban | 4.31% |
| 24 | Nolan et al., 2008 [24] | Cohort study | Airborne | Small airborne particles (dust, aerosols, small respiratory droplets) | Nosocomial | North America | Urban | 2.54% |
| 25 | Quoilin et al., 2006 [25] | Cohort study | Contact | Skin-to-skin contact | Public | Europe | Unknown | 1.33% |
| 26 | Reid et al., 1983 [26] | Cohort study | Airborne | Small airborne particles (dust, aerosols, small respiratory droplets) | Nosocomial | Europe | Urban | 4.62% |
| 27 | Ridgway et al., 1993 [27] | Cohort study | Vehicle | Dry surfaces | Nosocomial | Europe | Unknown | 22.58% |
| 28 | Roos et al., 1988 [28] | Case report | Vector | Animals | Domestic | Europe | Urban | 77.78% |
| 29 | Sarangi et al., 1995 [29] | Case control study | Vehicle | Bedding, clothing and other fabric | Public | Europe | Urban | 14.29% |
| 30 | Stetler et al., 1985 [30] | Cohort study | Vehicle | Moist surfaces | Nosocomial | North America | Non-urban | 10.26% |
| 31 | Streeton et al., 1995 [31] | Cohort study | Contact | Skin-to-skin contact | Public | Oceania | Non-urban | 20.58% |
| 32 | Takahashi et al., 1998 [32] | Cross sectional study | Vehicle | Dry surfaces | Nosocomial | Asia | Urban | 79.59% |
| 33 | Valenzuela et al., 1990 [33] | Case report | Droplet | Large droplets | Public | North America | Urban | 100.00% |
| 34 | Verboon-Maciolek et al., 2000 [34] | Case report | Contact | Skin-to-skin contact | Nosocomial | Europe | Unknown | 71.43% |

**Supplementary Table 2. Risk of bias for included studies. Risk of bias was determined according to Critical Appraisal tool from the Joanna Briggs Institute [35].**

| Study ID | Clear inclusion criteria? | Condition measured in a standard, reliable way for all participants? | Valid identification methods of the condition for all participants? | Consecutive inclusion of participants? | Complete inclusion of participants? | Clear reporting of the demographics? | Clear reporting of clinical information? | Clear reporting of outcomes or follow up results? | Clear reporting of the presenting site/clinic demographics? | Appropriate statistical analysis? | Total yes answers: yellow (8-10), orange (5-7), red (0-4) |
| --- | --- | --- | --- | --- | --- | --- | --- | --- | --- | --- | --- |
| Asteberg 2006 [1] | Yes | Yes | Yes | Yes | Yes | No | Yes | Yes | No | No* | 7 |
| Barnham 1980 [2] | Yes | Yes | Yes | Yes | Yes | No | Yes | No | No | No | 6 |
| Barnham 1981 - ob1 [3] | Yes | Yes | Yes | Yes | Yes | No | No | No | No | No | 5 |
| Barry 1997 [4] | Yes | Yes | Yes | Yes | Yes | No | Yes | No | No | Yes | 7 |
| Berkelman 1982 [5] | Yes | Yes | Yes | Yes | Yes | No | Yes | Yes | No | Yes | 8 |
| Berkley 1986 [6] | Yes | Yes | Yes | Yes | Yes | No | Yes | No | No | Yes | 7 |
| Bingen 1992 [7] | Yes | Yes | Yes | Yes | Yes | Yes | Yes | Yes | No | No | 8 |
| Colling 1980 [8] | Yes | No | Yes | Yes | Yes | Yes | Yes | Yes | Yes | No | 8 |
| Factor 1997 [9] | N/A - partially published (abstract) | | | | | | | | | | |
| Falck 1992 [10] | Yes | Yes | Yes | Yes | Yes | Yes | Yes | Yes | Yes | No | 9 |
| Falck 1996 [11] | Yes | Yes | Yes | Yes | No | No | No | No | No | No | 4 |
| Fehrs 1987 [12] | Yes | Yes | Yes | Yes | Yes | Yes | No | No | Yes | No* | 7 |
| Gisser 2001 [13] | Yes | Yes | Yes | Yes | Yes | Yes | Yes | Yes | No | No | 8 |
| Gordon 1994 [14] | Yes | Yes | Yes | Yes | Yes | No | Yes | No | No | No | 6 |
| Kemble 2013 [15] | Yes | Yes | Yes | Yes | Yes | Yes | No | No | No | Yes | 7 |
| Laustrup 2003 [16] | Yes | Yes | Yes | Yes | No | No | Yes | Yes | No | No | 6 |
| Lehtonen 1987 [17] | Yes | Yes | Yes | Yes | Yes | No | Yes | No | No | No* | 6 |
| Mahida 2014 [18] | Yes | Yes | Yes | Yes | Yes | Yes | Yes | No | No | No | 7 |
| Mahida 2018 [19] | Yes | Yes | Yes | Yes | Yes | Yes | Yes | Yes | No | No* | 8 |
| Manalo 2002 [20] | Yes | Yes | Yes | Yes | Yes | Yes | Yes | Yes | No | No | 8 |
| Mastro 1990 [21] | Yes | Yes | Yes | Yes | Yes | Yes | Yes | Yes | No | No* | 8 |
| Matsumoto 1999 [22] | Yes | Yes | Yes | Yes | No | No | No | No | No | No | 4 |
| McGregor 1984 [23] | Yes | Yes | Yes | Yes | Yes | No | Yes | No | No | No | 6 |
| Nolan 2008 [24] | Yes | Yes | Yes | Yes | Yes | No | Yes | No | No | No | 6 |
| Quoilin 2006 [25] | Yes | Yes | Yes | Yes | Yes | No | No | No | Yes | No | 6 |
| Reid 1983 [26] | Yes | Yes | Yes | Yes | Yes | Yes | Yes | Yes | Yes | No | 9 |
| Ridgway 1993 - ob2 [27] | Yes | Yes | Yes | Yes | Yes | No | Yes | Yes | No | No | 7 |
| Roos 1988 [28] | Yes | Yes | Yes | Yes | Yes | No | No | Yes | No | No | 6 |
| Sarangi 1995 [29] | Yes | Yes | Yes | Yes | Yes | No | No | No | No | Yes | 6 |
| Stetler 1985 - ob2 [30] | Yes | Yes | Yes | Yes | Yes | No | No | Yes | No | Yes | 7 |
| Streeton 1995 [31] | Yes | Yes | Yes | Yes | Yes | Yes | Yes | No | Yes | No* | 8 |
| Takahashi 1998 [32] | Yes | Yes | Yes | Yes | Yes | No | No | Yes | No | No | 6 |
| Valenzuela 1991 [33] | Yes | Yes | Yes | Yes | Yes | Yes | Yes | Yes | No | No | 8 |
| Verboon-Maciolek 2000 [34] | Yes | Yes | Yes | Yes | Yes | Yes | Yes | Yes | No | No | 8 |

* indicates downgraded due to uncertainty of the numerator and denominator used to calculate the attack rate. Ob = outbreak

**References (Supplementary Tables)**

[1] Asteberg I, Andersson Y, Dotevall L, Ericsson M, Darenberg J, Henriques-Nordmark B, et al. A food-borne streptococcal sore throat outbreak in a small community. Scand J Infect Dis. 2006;38(11-12):988-94.

[2] Barnham M, Kerby J, Skillin J. An outbreak of streptococcal infection in a chicken factory. J Hyg (Lond). 1980;84(1):71-5.

[3] Barnham M, Kerby J. Skin sepsis in meat handlers: observations on the causes of injury with special reference to bone. J Hyg (Lond). 1981;87(3):465-76.

[4] Centers for Disease Control and Prevention (CDC). Outbreak of invasive group A *Streptococcus* associated with varicella in a childcare center -- Boston, Massachusetts, 1997. MMWR Morb Mortal Wkly Rep. 1997;46(40):944-8.

[5] Berkelman RL, Martin D, Graham DR, Mowry J, Freisem R, Weber JA, et al. Streptococcal wound infections caused by a vaginal carrier. JAMA. 1982;247(19):2680-2.

[6] Berkley SF, Rigau-Pérez JG, Facklam R, Broome CV. Foodborne streptococcal pharyngitis after a party. Public Health Rep. 1986;101(2):211-5.

[7] Bingen E, Denamur E, Lambert-Zechovsky N, Boissinot C, Brahimi N, Aujard Y, et al. Mother-to-infant vertical transmission and cross-colonization of *Streptococcus pyogenes* confirmed by DNA restriction fragment length polymorphism analysis. J Infect Dis. 1992;165(1):147-50.

[8] Colling A, Kerr I, Maxted WR, Widdowson JP. Streptococcal infection in a Junior Detention Centre: a five-year study. J Hyg (Lond). 1980;85(3):331-41.

[9] Factor S, O'Brien K, Matthews K, Jackson D, Brudzinski L, Nydam D, et al. Outbreak of Group A *Streptococcus* (GAS) Associated with Varicella in a Day Care Center. In Abstracts of the 1997 Annual IDSA Meeting. Clin Infect Dis. 1997;25(2):410.

[10] Falck G, Kjellander J. Outbreak of group A streptococcal infection in a day-care center. Pediatr Infect Dis J. 1992;11(11):914-9.

[11] Falck G. Group A streptococcal skin infections after indoor association football tournament. Lancet. 1996;347(9004):840-1.

[12] Fehrs LJ, Flanagan K, Kline S, Facklam RR, Quackenbush K, Foster LR. Group A beta-hemolytic streptococcal skin infections in a US meat-packing plant. JAMA. 1987;258(21):3131-4.

[13] Gisser JM, Fields MC, Pick N, Moses AE, Srugo I. Invasive group A *Streptococcus* associated with an intrauterine device and oral sex. Sexually transmitted diseases. 2002;29(8):483-5.

[14] Gordon G, Dale BA, Lochhead D. An outbreak of group A haemolytic streptococcal puerperal sepsis spread by the communal use of bidets. Br J Obstet Gynaecol. 1994;101(5):447-8.

[15] Kemble SK, Westbrook A, Lynfield R, Bogard A, Koktavy N, Gall K, et al. Foodborne outbreak of group A *Streptococcus* pharyngitis associated with a high school dance team banquet--Minnesota, 2012. Clin Infect Dis. 2013;57(5):648-54.

[16] Laustrup HK, Justesen US, Pedersen C. Household transmission of invasive group A *Streptococcus* with necrotizing fasciitis. Scand J Infect Dis. 2003;35(6-7):414-5.

[17] Lehtonen OP, Kero P, Ruuskanen O, Gaworzewska ET, Hollo O, Erkkola R, et al. A nursery outbreak of group A streptococcal infection. J Infect. 1987;14(3):263-70.

[18] Mahida N, Beal A, Trigg D, Vaughan N, Boswell T. Outbreak of invasive group A *Streptococcus* infection: contaminated patient curtains and cross-infection on an ear, nose and throat ward. J Hosp Infect. 2014;87(3):141-4.

[19] Mahida N, Prescott K, Yates C, Spencer F, Weston V, Boswell T. Outbreak of invasive group A streptococcus: investigations using agar settle plates detect perineal shedding from a healthcare worker. J Hosp Infect. 2018;100(4):e209-e15.

[20] Manalo R, Mirza H, Opal S. *Streptococcus pyogenes* tuboovarian abscess: a potential sexually transmitted disease? Sexually transmitted diseases. 2002;29(10):606-7.

[21] Mastro TD, Farley TA, Elliott JA, Facklam RR, Perks JR, Hadler JL, et al. An outbreak of surgical-wound infections due to group A streptococcus carried on the scalp. N Engl J Med. 1990;323(14):968-72.

[22] Matsumoto M, Miwa Y, Matsui H, Saito M, Ohta M, Miyazaki Y. An outbreak of pharyngitis caused by food-borne group A *Streptococcus*. Jpn J Infect Dis. 1999;52(3):127-8.

[23] McGregor J, Ott A, Villard M. An epidemic of "childbed fever". Am J Obstet Gynecol. 1984;150(4):385-8.

[24] Nolan L, Schertzberg R, Wilson-Clark S, McGeer A, Pellizzari R, Steingart C. Outbreak of invasive group A streptococcal disease in two hospitals, Ontario, 2003. Can Commun Dis Rep. 2008;34(4):8-19.

[25] Quoilin S, Lambion N, Mak R, Denis O, Lammens C, Struelens M, et al. Soft tissue infections in Belgian rugby players due to *Streptococcus pyogenes emm* type 81. Euro Surveill. 2006;11(12):E061221.2.

[26] Reid RI, Briggs RS, Seal DV, Pearson AD. Virulent *Streptococcus pyogenes*: outbreak and spread within a geriatric unit. J Infect. 1983;6(3):219-25.

[27] Ridgway EJ, Allen KD. Clustering of group A streptococcal infections on a burns unit: important lessons in outbreak management. J Hosp Infect. 1993;25(3):173-82.

[28] Roos K, Lind L, Holm SE. Beta-haemolytic streptococci group A in a cat, as a possible source of repeated tonsillitis in a family. Lancet. 1988;2(8619):1072.

[29] Sarangi J, Rowsell R. A nursing home outbreak of group A streptococcal infection: case control study of environmental contamination. J Hosp Infect. 1995;30(2):162-4.

[30] Stetler HC, Garbe PL, Dwyer DM, Facklam RR, Orenstein WA, West GR, et al. Outbreaks of group A streptococcal abscesses following diphtheria-tetanus toxoid-pertussis vaccination. Pediatrics. 1985;75(2):299-303.

[31] Streeton CL, Hanna JN, Messer RD, Merianos A. An epidemic of acute post-streptococcal glomerulonephritis among aboriginal children. J Paediatr Child Health. 1995;31(3):245-8.

[32] Takahashi A, Yomoda S, Tanimoto K, Kanda T, Kobayashi I, Ike Y. *Streptococcus pyogenes* hospital-acquired infection within a dermatological ward. J Hosp Infect. 1998;40(2):135-40.

[33] Valenzuela TD, Hooton TM, Kaplan EL, Schlievert P. Transmission of 'toxic strep' syndrome from an infected child to a firefighter during CPR. Annals of emergency medicine. 1991;20(1):90-2.

[34] Verboon-Maciolek MA, Krediet TG, van Ertbruggen I, Gerards LJ, Fleer A. Severe neonatal group A streptococcal disease. Eur J Pediatr. 2000;159(6):450-2.

[35] Moola S, Munn Z, Tufanaru C, Aromataris E, Sears K, Sfetcu R, et al. Systematic reviews of etiology and risk. In: Aromataris E, Munn Z, editors. Joanna Briggs Institute Reviewer's Manual: The Joanna Briggs Institute; 2017. p. 1-6.
